# Time-Dependent Alterations of MMPs, TIMPs and Tendon Structure in Human Achilles Tendons after Acute Rupture

**DOI:** 10.3390/ijms18102199

**Published:** 2017-10-20

**Authors:** Susann Minkwitz, Aysha Schmock, Alper Kurtoglu, Serafeim Tsitsilonis, Sebastian Manegold, Britt Wildemann, Franka Klatte-Schulz

**Affiliations:** 1 Julius Wolff Institute, Center for Musculoskeletal Surgery, Charité—Universitätsmedizin Berlin, Corporate Member of Freie Universität Berlin, Humboldt-Universität zu Berlin, and Berlin Institute of Health, 13353 Berlin, Germany; Susann.Minkwitz@charite.de (S.M.); Aysha.Schmock@charite.de (A.S.); Alper.Kurtoglu@charite.de (A.K.); Serafeim.Tsitsilonis@charite.de (S.T.); Sebastian.Manegold@charite.de (S.M.); Franka.Klatte@charite.de (F.K.-S.); 2 Berlin-Brandenburg Center for Regenerative Therapies, Charité—Universitätsmedizin Berlin, Corporate Member of Freie Universität Berlin, Humboldt-Universität zu Berlin, and Berlin Institute of Health, 13353 Berlin, Germany

**Keywords:** tendon healing, human, matrix metalloproteinases, tissue inhibitors of metalloproteinases, ECM composition

## Abstract

A balance between matrix metalloproteinases (MMPs) and their inhibitors (TIMPs) is required to maintain tendon homeostasis. Variation in this balance over time might impact on the success of tendon healing. This study aimed to analyze structural changes and the expression profile of MMPs and TIMPs in human Achilles tendons at different time-points after rupture. Biopsies from 37 patients with acute Achilles tendon rupture were taken at surgery and grouped according to time after rupture: early (2–4 days), middle (5–6 days), and late (≥7 days), and intact Achilles tendons served as control. The histological score increased from the early to the late time-point after rupture, indicating the progression towards a more degenerative status. In comparison to intact tendons, qRT-PCR analysis revealed a significantly increased expression of MMP-1, -2, -13, TIMP-1, COL1A1, and COL3A1 in ruptured tendons, whereas TIMP-3 decreased. Comparing the changes over time post rupture, the expression of MMP-9, -13, and COL1A1 significantly increased, whereas MMP-3 and -10 expression decreased. TIMP expression was not significantly altered over time. MMP staining by immunohistochemistry was positive in the ruptured tendons exemplarily analyzed from early and late time-points. The study demonstrates a pivotal contribution of all investigated MMPs and TIMP-1, but a minor role of TIMP-2, -3, and -4, in the early human tendon healing process.

## 1. Introduction

Acute Achilles tendon ruptures are one of the most common sports-related injuries and incidences have increased over the previous decades [1]. Despite the progress observed in the available surgical techniques over the years, chronic functional impairments are often observed in the patients [2]. The slow and complex process of tendon healing often results in the formation of scar tissue of inferior quality with a decreased capacity for bearing high biomechanical forces [3]. The parameters for the low healing potential of tendon ruptures and the underlying cellular and molecular background remain mostly unknown. Detrimental changes in the expression of genes regulating the collagen balance, such as matrix metalloproteinases (MMPs) and their natural inhibitors, the tissue inhibitors of metalloproteinases (TIMPs), have been hypothesized to account for the impaired healing ability [4,5,6,7,8]. The balance between MMPs and TIMPs is therefore required to maintain tendon homeostasis [9].

MMPs are a large family of proteolytic enzymes, which degrade all components of the extracellular matrix (ECM) of the tendon and are antagonized by TIMPs [7,10]. They are classified in groups related to their function: collagenases such as MMP-1, MMP-8, MMP-13, and MMP-18 degrade collagens, the most important component of the tendon matrix [5,11]. The gelatinases MMP-2 and MMP-9 cleave smaller collagen fragments and gelatin. The stromelysines MMP-3, MMP-10, MMP-11, the matrilysins MMP-7 and MMP-26, and the metalloelastase MMP-12 degrade primarily glycoproteins and proteoglycans [10,11]. The last group consists of membrane-type MMPs (MMP-14 to -17), which have a regulatory function and can activate other MMPs [12,13]. MMP activity is regulated by the formation of complexes with their four endogenous antagonists (TIMP-1, -2, -3, -4). The inhibitory effects of the TIMPs are overlapping, since they are not specific to a single MMP group [14]. TIMPs are able to interfere with the active, as well as latent, form of MMPs and in addition to their inhibitory activities they also have promoting functions. TIMPs play an important role in the regulation of cell growth, differentiation, apoptosis, and angiogenesis [15,16].

Until now, the processes and timing of matrix remodeling and formation that occur after tendon rupture have not been completely understood in humans. During tendon healing, the typical processes are similar to other soft tissues: inflammation within the first days, followed by a reparative/proliferative phase, the remodeling/consolidation phase, and at the end the maturation of the tissue [17]. Hence, it was the aim of the present study to detect time-dependent changes in the expression of tendon-related MMPs, TIMPs, and ECM markers, as well as the structure of Achilles tendons after ruptures at different post-rupture time-points in order to gain basic knowledge about early human Achilles tendon healing.

## 2. Results

### 2.1. Histological Analysis

**Figure 1:**
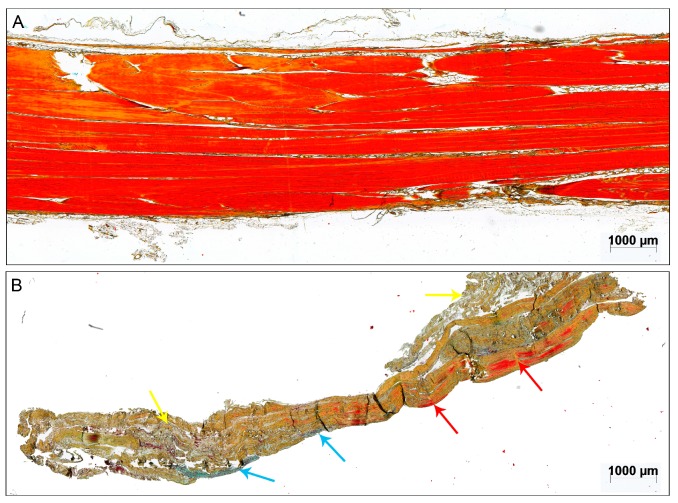
Exemplary sample of (**A**) an intact cadaveric tendon and (**B**) a 3-days-old acute ruptured Achilles tendon stained with Movat Pentachrome (MP). The red color of the otherwise brownish collagen fibers indicates mature collagen [18]. The red arrows point to intact parallel-aligned collagen fibers in the ruptured tendon. The blue arrows point to glycosaminoglycan (GAG)-rich areas and the yellow arrows mark unorganized tissue. Scale bar: 1000 μm.

The intact cadaveric tendons showed aligned tendon fibers of mature collagen without visible glycosaminoglycan (GAG)-content and fat tissue and were all scored with 0 points (Figure 1A). Vessels were only detected between the collagen fiber bundles in the endotenon (data not shown). The structure of the ruptured tendon tissue samples was mainly inhomogeneous. Most of the tendons lost their tendon architecture completely (exemplary sample Figure 1B).

**Figure 2:**
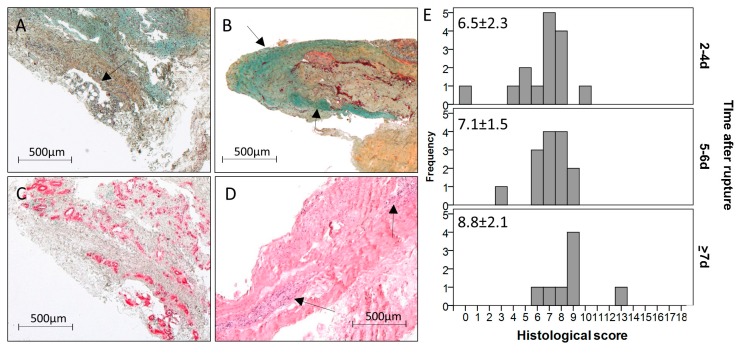
Exemplary pictures of acute ruptured Achilles tendon samples showing (**A**) fat tissue (black arrow, MP staining, 10-days-old rupture), (**B**) high GAG content stained in turquoise (black arrows, MP staining, 2-days-old rupture), (**C**) massive vascularity visualized by α-SMA staining (10-days-old rupture), and (**D**) large cell clusters visualized by H&E staining (black arrows, 5-days-old rupture). Scale bar: 500 μm. (**E**) Histogram showing the frequency of points of the histological score over the three time-point groups. The score increased from the early to the late time point (*p* = 0.023) and from the middle to the late time point (*p* = 0.050) after rupture. However, differences did not stay significant after Bonferroni–Holm correction.

The amount of aligned collagen tended to decrease over time post rupture. In two of the 37 samples, fat tissue was detectable (Figure 2A), both in the late time-point group. The GAG-content (Figure 2B) was present in 11 samples and did not change over time. The cellularity varied from a normal cell distribution, as seen in intact tendons, to clusters of varying sizes (Figure 2D), which increased over time (2–4 days: 0.9 points, 5–6 days: 0.9 points, ≥7 days: 1.6 points). The same trend could be observed for the vascularity (Figure 2C; 2–4 days: 0.5 points, 5–6 days: 0.6 points, ≥7 days: 1.1 points). Variations in the leukocyte infiltration were not detectable with the utilized stainings. Taken together, the histological score (Figure 2E) was significantly higher in the late time-point group compared to the early time-point group (2–4 days: 6.5 points, ≥7 days: 8.8 points; *p* = 0.023) and to the middle time-point group (2–4 days: 7.1 points, ≥7 days: 8.8 points; *p* = 0.050). However, differences did not stay significant after Bonferroni–Holm correction.

### 2.2. MMP, TIMP, and Collagen Expression

**Figure 3:**
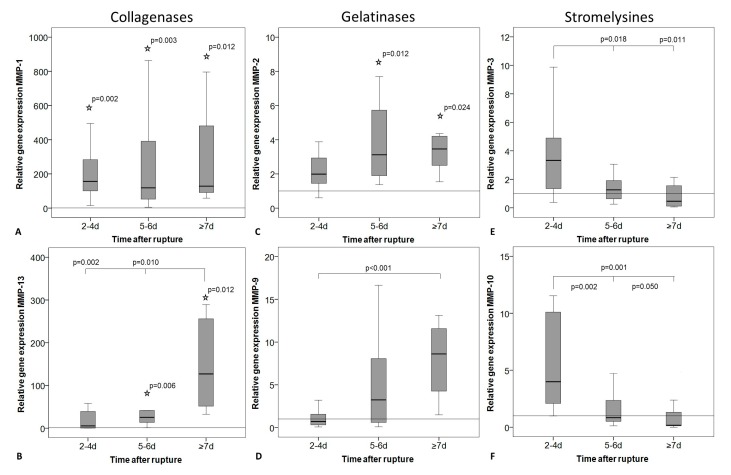
Relative gene expression of matrix metalloproteinases (MMPs) in intact tendon tissue (horizontal line, value = 1) and in tendon tissue of early (2–4 days), middle (5–6 days), and late (≥7 days) time after rupture. Measures of qRT-PCR were normalized to the expression of the house keeping gene 18S-rRNA using the Δ*C*_t_ method with efficiency correction, given as fold change to the intact group (horizontal line, value = 1), and represented as box plot graphs. Significant differences to the intact group are marked with a star and time-related differences with a spanning line. (**A**) MMP-1, (**B**) MMP-13, and (**C**) MMP-2 expression was significantly lower in the intact tendons compared to the ruptured tendons from certain time-points marked with a star. (**A**) MMP-1 and (**C**) MMP-2 expression was not regulated over time, while (**D**) MMP-9 and (**B**) MMP-13 significantly increased with the time after rupture and (**E**) MMP-3 and (**F**) MMP-10 decreased.

Gene expression of the collagenases MMP-1 and MMP-13 and the gelatinase MMP-2 was higher in the ruptured tendons compared to the intact tendons (Figure 3A–C). The expression of MMP-3, MMP-9, and MMP-10 showed no significant differences between the intact and the ruptured groups. However, significant differences were present before *p*-value adjustment for MMP-9 (late group, *p* = 0.024) and MMP-10 (early group, *p* = 0.039). No changes in MMP-1 expression could be observed over time in the ruptured tendons (Figure 3A). The expression of MMP-2, MMP-9, and MMP-13 increased with the time after rupture (Figure 3B–D). However, for MMP-2, the differences were not significant after Bonferroni–Holm correction (*p* = 0.028). The expression of the stromelysines MMP-3 and MMP-10 was significantly decreased with the time after rupture (Figure 3E,F).

**Figure 4:**
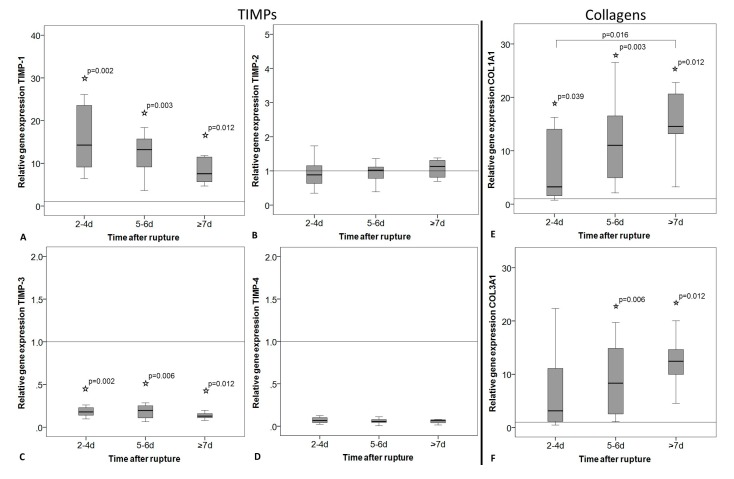
Relative gene expression of tissue inhibitors of metalloproteinases (TIMPs) and collagens in intact tendon tissue (horizontal line, value = 1) and in tendon tissue of early (2–4 days), middle (5–6 days), and late (≥7 days) time after rupture. Measures of qRT-PCR were normalized to the expression of the house keeping gene 18S-rRNA using the ΔCt method with efficiency correction, given as fold change to the intact group (horizontal line, value = 1), and represented as box plot graphs. Significant differences to the intact group are marked with a star and time-related differences with a spanning line. (**A**) TIMP-1 expression was significantly lower, whereas (**C**) TIMP-3 expression was significantly higher in the intact compared to the ruptured tendons. (**A**–**D**) In general, TIMP expression did not change significantly over time. (**E**) COL1A1 and **(F**) COL3A1 expression was significantly increased in ruptured tendons compared to intact tendons. COL1A1 expression significantly increased with the time after rupture from the early to the late time point group.

Gene expression of TIMP-1 was significantly higher in the ruptured tendons compared to the intact group (Figure 4A) and decreased over time without significant differences after Bonferroni–Holm correction (*p* = 0.034). The expression of TIMP-3 showed a significantly higher level in the intact tendons compared to the ruptured ones, but no changes over time (Figure 4C). TIMP-2 and -4 expression showed no alteration over time or between intact and ruptured samples (Figure 4B,D). Even if the TIMP-4 expression was strongly reduced in the ruptured tendons, this was not significantly different to the intact tendons due to the high variability within the intact group. The expression of Col1A1 and COL3A1 was significantly increased in the ruptured tendons compared to the intact tendons. A significant increase in the expression of COL1A1 was additionally found over time after rupture (Figure 4E). Also, COL3A1 increased, but without significant differences after *p*-value adjustment (Figure 4F, *p* = 0.034). The COL1A1/COL3A1 ratio did not change between intact and ruptured tendons or over time.

### 2.3. Immunohistochemistry

**Figure 5:**
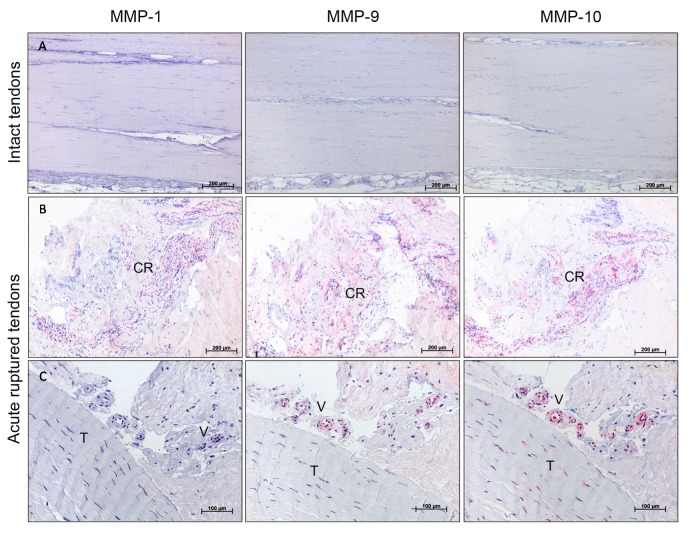
Exemplary results of immunohistological staining for MMP-1, MMP-9, and MMP-10 of representative areas of intact controls versus acute ruptured Achilles tendons (3-days-old ruptures). (**A**) Intact cadaveric tendons showed no MMP staining. Scale bar: 200 μm. (**B**) Positive MMP staining of cell-rich areas (CR). Scale bar: 200 μm. (**C**) Positive MMP-10 and slightly positive MMP-1 and MMP-9 staining of tenocytes (T) and positive MMP-9 and -10 and slightly positive MMP-1 staining around blood vessels (V). Scale bar: 100 μm.

To visualize the localization of MMPs in tendon tissue, immunohistochemistry was performed for selected MMPs where the gene expression was highly regulated, as shown in Figure 3. The exemplary, descriptive analysis of MMP-1, MMP-9, and MMP-10 showed no detectable staining in the aligned collagen fibers of the intact cadaveric tendon tissue (Figure 5, top row). In the ruptured tendons from all time-points, a positive signal was mostly present in cell-rich areas and was related to blood vessel formation. Tenocytes were mainly MMP-positive at the tendon edges in proximity to areas undergoing tendon remodeling, but to a lesser extent in visually healthy parts of the tendons. In general, MMP-10 showed the strongest staining of tenocytes (Figure 5, last column).

### 2.4. Correlation Analysis

For the conventional histological analysis, the time after rupture correlated weakly with the fat content (r_s_ = 0.335, *p* = 0.043). For the expression analysis, the time after rupture correlated positively with the expression of the gelatinases (MMP-2: r_s_ = 0.375, *p* = 0.022; MMP-9: r_s_ = 0.569, *p* < 0.001) and MMP-13 (r_s_ = 0.547, *p* < 0.001), and negatively with the stromelysines (MMP-3: r_s_ = −0.522, *p* = 0.001; MMP-10: r_s_ = −0.745, *p* < 0.001) and TIMP-1 (r_s_ = −0.467, *p* = 0.004). Furthermore, a moderate correlation was observed for the time after rupture with the COL1A1 (r_s_ = 0.459, *p* = 0.004) and COL3A1 (r_s_ = 0.375, *p* = 0.022) expression.

Several weak to moderate correlations were observed for the expression of the MMPs and TIMPs with the histological data. Disturbed tendon architecture correlated positively with the MMP-1 expression (r_s_ = 0.357, *p* = 0.030) and COL1A1 to COL3A1 ratio (r_s_ = 0.392, *p* = 0.016). The amount of aligned collagen (actual %-values) correlated negatively with the expression of MMP-1 (r_s_ = −0.501, *p* = 0.002), MMP-2 (r_s_ = −0.432, *p* = 0.008), and MMP-9 (r_s_ = −0.362, *p* = 0.027). Positive correlations were found for the GAG-content with the MMP-13 (r_s_ = 0.326, *p* = 0.049), TIMP-2 (r_s_ = 0.383, *p* = 0.019), and TIMP-4 (r_s_ = 0.462, *p* = 0.004) expression. Moderate correlations were observed for the vascularity with the TIMP-4 expression (r_s_ = 0.539, *p* = 0.001). The total histological score correlated positively with the expression of MMP-9 (r_s_ = 0.437, *p* = 0.007), MMP-13 (r_s_ = 0.495, *p* = 0.002), TIMP-2 (r_s_ = 0.381, *p* = 0.020) and TIMP-4 (r_s_ = 0.408, *p* = 0.012), COL1A1 (r_s_ = 0.413, *p* = 0.011), and COL3A1 (r_s_ = 0.432, *p* = 0.008).

## 3. Discussion

This present study shows for the first time that the expression profile of MMPs and TIMPs, as well as the structural tendon properties, are altered over time during the tendon healing process after acute rupture. MMP and TIMP alterations were reported previously in traumatic or tendinopathic versus intact tendons or were determined over the healing process in animal models, and are discussed later. However, no previous study exists that has analyzed the expression profile of MMPs and TIMPs and the tendon structure at different time-points after acute rupture in a human tendon healing model. A comparable animal healing model, harvesting ligament biopsy samples at different time-points after Anterior Cruciate Ligament (ACL) transection, was described previously [4].

The histological analysis, using a modified Movin score [19], revealed an increased histological score from the early (2–4 days) and middle (5–6 days) to the late (≥7 days) phase after rupture, showing a more abnormal tendon structure. This might indicate alterations in a degenerative direction, possibly due to the proteolytic activity of the collagenases (MMP-1 and MMP-13) and gelatinases (MMP-2 and MMP-9). This suggestion could be supported by the correlation analysis, where the disturbed tendon architecture and the amount of aligned collagen correlated positively with the MMP-1, -2, and -9 expression. The total histological score additionally revealed a positive correlation with the MMP-9 and -13, as well as TIMP-2 and TIMP-4, expression, underlining the relationship of MMPs and TIMPs with alterations in tendon structure. Exemplary immunohistochemical staining of MMP-1, -9, and -10 showed the presence of MMPs in the ruptured tendon, whereas no MMPs were detected in the intact cadaveric tendons, likewise indicating their active role in the healing process.

The comparison of the present results to other studies is mostly limited to the comparison of intact versus ruptured tendons, due to the lack of information regarding alterations in MMP and TIMP expression over time. The expression of the collagenases MMP-1 and -13 as well as the gelatinase MMP-2 was higher in ruptured compared to intact tendon samples. This indicates that alterations start at a very early time-point of less than 2 days post rupture and might initiate degradation and remodeling of the tendon ECM. The present findings confirm several other studies, where the expression or activity of MMP-1, -9, and -13 increased in ruptured tendons compared to healthy controls [20,21,22,23,24]. For MMP-2, contradictory findings have been reported, with an increased [25] as well as decreased [22] MMP-2 activity in ruptured compared to healthy tendons. The increase in the collagenases and the gelatinases during the healing process might in vivo lead to a strong increase in degradation of the tendon ECM, especially of collagens and collagen fragments, which is necessary for tendon modeling and remodeling. Regarding the MMP inhibitors, TIMP-1 expression was higher and TIMP-3 expression was lower in the ruptured compared to the intact tendons, indicating a very early regulation of these TIMPs during tendon healing. In line with the present findings, TIMP-1 was upregulated with Achilles tendon rupture in other studies [20,25]. Furthermore, it was described that TIMP-1 inhibits excessive degradation of the ECM by MMP-2 [25] and therefore might be found to be strongly increased after rupture. TIMP-2, -3, and -4 were found in decreased amounts in rotator cuff ruptures compared to healthy controls [20,23]. The absent or negative regulations of TIMP-2, -3, and -4 in the present study indicate their secondary role in the early human tendon healing process.

It has been previously shown that the age and degenerative status of Supraspinatus tendons is related to an increased expression and secretion of MMP-2, MMP-9, MMP-13, as well as TIMP-1, -2, and -3 in tenocytes [26]. Also, other authors have found a relationship between altered expression of MMPs and TIMPs and chronic tendon pathologies [6,21,27,28,29,30]. The increase/decrease of MMPs and TIMPs, as seen in chronic tendon pathologies, might indicate that with increasing time after rupture the tendon tissue at the rupture site is moving in a degenerative direction. This could also be confirmed by the histological analysis, showing a more abnormal tendon in the later time-point group. Whether these alterations are taking place due to the normal tendon healing process, or if these changes also have a negative impact on the tendon healing outcome, cannot be clarified within the present study. However, a relationship between MMPs and the healing outcome after Rotator Cuff reconstruction has already been proven in a clinical study where the increase of MMP-1 and MMP-9 expression at the time of surgery correlated with failed healing more than 6 months after surgery [31]. Agres et al. demonstrated altered tendon properties with a higher tendon stiffness and elongation compared to an intra-patient control two to six years after Achilles tendon reconstruction [32]. The reason for these sustained tendon alterations is unclear, but might be due to an ineffective remodeling process possibly triggered by MMP/TIMP regulation. A recent study found no effect of early surgical intervention after Achilles tendon rupture (≤24 h, 24–48 h, ≥48 h–1 week) in respect to muscle strength and several outcome scores one year after surgery [33]. With the strongest MMP/TIMP imbalance observed in the group operated on 7–10 days after rupture, it would indicate that 7 days might be the critical border for clinical intervention.

Detailed information about the time-dependent alterations of MMPs and TIMPs in human tendon healing is not available. Presently, an increased expression of MMP-13, MMP-2 (confirmed by correlation analysis), and MMP-9 as well as COL1A1 and COL3A1 (correlation analysis) was found over time after rupture, whereas MMP-3 and MMP-10 and TIMP-1 (correlation analysis) decreased. Interestingly, the TIMPs showed no significant alterations over time by group comparisons. Comparable results were described previously after ACL transection in minipigs, where MMP-13 as well as COL1A3 and COL3A1 increased over time [4]. In rat and mouse healing models, MMP-9 and MMP-13 participated in the early phase of tissue degradation and expression decreased afterwards, whereas MMP-2 and MMP-3 expression remained high until the later remodeling process [34,35]. The participation of all mentioned MMPs in the healing process could be confirmed by the present results. However, the continuous high expression of MMP-3 is in contrast to the present findings of a decreased expression at the later time-point group. However, in the present study, MMP and TIMP expression was only analyzed until day 10 post rupture. Therefore, a comparison to the later remodeling phases was not possible. It is unclear if MMP-3 plays a positive or negative role in the tendon healing process. Based on its known function in activating other MMPs, it has been suggested that a decreased expression of MMP-3 leads to an incorrect remodeling process [22]. In an immunohistological study on rabbit Supraspinatus tendons, MMP-2 and TIMP-1 were found 7 and 14 days after rupture and decreased afterwards. In contrast, TIMP-2 was constitutively expressed during the whole healing period [25], which is comparable to the present findings. Animal studies showed that COL3A1 expression is mainly induced in early tendon healing, which leads to tendon scar formation, whereas an increase in COL1A1 seems to play a minor role [34,36]. However, this is in contrast to the present results of a similar increasing expression pattern of COL1A1 and COL3A1 and no change in COL1A1 to COL3A1 ratio over time. The upregulation of COL1A1 might be a result of the increased MMP activity to overcome the excessive collagen degradation.

Taken together, all MMPs as well as TIMP-1 and -3 were regulated between intact and ruptured tendon samples and/or over time, which indicates their active contribution in the early human tendon healing process. When looking at the alterations found over time, the increased expression of MMP-2, MMP-9, and MMP-13 together with the decrease of the more regulatory MMP-3 and -10 and the simultaneous lack of regulation of TIMP expression over time might lead to a highly imbalanced MMP/TIMP ratio in vivo. This in turn might result in an incorrect remodeling process. Even if this is currently very speculative, the composition of MMPs/TIMPs in the ruptured tendon might some day be used as biomarker for the healing outcome. However, this requires further evaluation and a correlation to the healing outcome, which was not possible within the present study. Today, MMPs are already discussed as biomarkers for diagnosis and prognosis in various cancer types [37], but there are a several limitations [38].

The limitations of the present study are as follows. The first is the low number of intact tendons from patients, which were used as control for RNA analysis. This is caused by the limited availability of intact tendon samples due to the strict ethical regulations. However, the focus of the present study was to evaluate acute ruptured Achilles tendons at different time-points post rupture, and distinct differences to the intact samples could be demonstrated. In addition, the MMP proteins were localized by immunohistochemistry in the tendon tissue sections in a descriptive manner. Due to limited sample material, a quantification of the protein and the validation of the MMP activity were not possible. However, it was previously shown that the mRNA and protein levels of MMPs correlate well [7,10,11]. The last limitation to mention is the limited time frame of 2 to 10 days. It would be interesting to also look at later time-points to gain more information regarding the remodeling process in which the MMPs might also play a crucial rule.

## 4. Materials and Methods

### 4.1. Human Achilles Tendon Samples

Intraoperative samples of acute ruptured Achilles tendons were collected directly from the rupture site from 37 patients undergoing minimal-invasive Achilles tendon healing between May 2014 and August 2016. The surgery was performed 2 to 10 days after rupture, which represents the inflammatory and the early proliferative phase of the tendon healing cascade. Patients were grouped into early (2–4 days after rupture, *n* = 15, mean: 3.2 ± 0.8 days), middle (5–6 days after rupture, *n* = 14, mean: 5.6 ± 0.5 days), and late (≥7 days after rupture, *n* = 8, mean: 8.3 ± 1.0 days) time-points. The three female and 34 male patients had a mean age of 38.2 ± 9.3 years and a body mass index (BMI) of 25.2 ± 3.0, which was equally distributed over the groups (See Table 1). A total of 32 of the 37 patients were recreational sportsmen. Patients who suffered from Achilles tendinopathy prior to rupture were excluded as well as patients who had received medication that might influence tendon structure (cortisone, antibiotics, and anabolic steroids). The acute ruptured tendon samples were divided for Quantitative Real-Time PCR (qRT-PCR) and a histological analysis. Additionally, intraoperative samples of intact tendons were collected from one female and two male patients (mean age of 39 years) undergoing surgery not related to primary tendon pathology (tendon elongation) and served as controls for gene expression analysis. Moreover, intact cadaveric Achilles tendon tissue (one male/four female, with the body donated to a university anatomy program with permission to use for a research purpose) was used for the histological analysis. The study was approved by the local Institutional Review Board (IRB) (EA2/074/14), and all patients gave their written informed consent prior to surgery.

### 4.2. Histological Analysis

Tendon samples were washed twice in PBS and fixed in 4% paraformaldehyde solution for 24 h. Afterwards, they were rinsed in water and dehydrated automatically before being embedded into paraffin. Slices 4 μm thick were cut and stained with Hematoxylin Eosin (H&E), Movat Pentachrome (MP), and α-smooth muscle actin (αSMA, see details below) to histologically examine the grade of tendon damage using a modified Movin score [19]. The original Movin score was used to characterize longstanding achillodynia, a chronic pain syndrome of the Achilles tendon. As, in the present study, fresh ruptured Achilles tendon samples were analyzed, the score had to be modified. The following original parameters were evaluated: tendon architecture, GAG-content, cellularity, and vascularity. Compared to the original score, the amount of aligned collagen structure and fat content were additionally scored, whereas the rounding of nuclei, hyalinization, and collagen stainability was not evaluated. Each parameter was scored between 0 and 3, (0 = normal, 1 = slightly abnormal, 2 = abnormal, and 3 = markedly abnormal) to reach in total a maximum of 18 points (for the classifications see Table 2). The amount of aligned collagen and fat tissue was automatically quantified using ImageJ (ImageJ 1.44i, Wayne Rasband, National Institute of Health, Bethesda, MD, USA). The other characteristics were quantified visually by three blinded independent observers. Two observers scored the staining independently and the third observer decided in case of discrepancy. The interobserver variability was measured by Kendall’s Tau correlation analysis and revealed a substantial reliability between 0.616 and 0.734. The intraobserver variability showed a substantial to almost perfect reliability between 0.758 and 0.985.

### 4.3. Gene Expression Analysis

The tendon samples were stored in RNAlater (Qiagen, Hilden, Germany) solution directly after surgery for stabilization of tissue RNA. Afterwards, they were removed from RNAlater, frozen in liquid nitrogen, and then stored at −80 °C until RNA isolation was performed. The tissue homogenization was done using a precooled (liquid nitrogen) steel mortar system until a tissue powder was produced. The powder was incubated in 1 mL of Trifast peqGOLD (Peqlab, Erlangen, Germany) for 10 min at room temperature. Phase separation was performed by adding 200 µL chloroform, with vigorous shaking and incubation for 10 min at room temperature. After centrifugation at 14,000× *g* for 10 min at room temperature, the upper aqueous phase was mixed with 500 µL 75% Ethanol. Afterwards, the RNA was further purified using the NucleoSpin RNA Kit (Macherey-Nagel, Düren, Germany) according to the manufacturer’s instructions. The quantity and purity of the received RNA was analyzed with the Nanodrop ND1000 system (Peqlab, Erlangen, Germany). Additionally, RNA integrity was analyzed exemplarily with the 2100 Bioanalyzer and the Agilent 6000 Pico Kit (Agilent), and reached values between 6.6 and 7.9, indicating the good quality of the RNA with only slight indication for degradation. The RNA was stored at −80 °C until cDNA synthesis. A total of 100 ng RNA was transcribed into cDNA with the qScript cDNA Supermix (Quanta Biosciences, Gaithersburg, MD, USA). qRT-PCR was performed with the SyBr Green Mastermix (Quanta biosciences) according to the manufacturer’s manual and the Light Cycler 480 System (Roche, Mannheim, Germany). All primer sequences were designed using Primer 3 software (Freeware; Available online: http://frodo.wi.mit.edu/primer3), and were produced by Tib Molbiol, Berlin, Germany (Primer sequences see Table 3). All primers were tested for amplification efficiency and the ΔCt method with efficiency correction was used to calculate the relative gene expression to the reference gene 18S-rRNA. Reference gene 18S rRNA was tested to be the most constant housekeeping gene over the three time-point groups compared to 60S ribosomal protein L13 (RPL13) and Hypoxanthine phosphoribosyl transferase (HPRT). MMPs and TIMP were selected based on previously published studies investigating their changes/regulation in tendon injuries and tenocytes [20,21,24,39].

### 4.4. Immunohistochemistry

Exemplary immunohistochemical stainings were performed for three samples per early and late time-point group. For immunohistochemistry, the tissue was incubated with the primary antibodies against αSMA (monoclonal mouse anti-human smooth muscle actin, 1:200, Dako, Jena, Germany, M0851), MMP-1 (monoclonal rabbit anti-human MMP-1, 1:50, Abcam, Cambridge, UK, ab52631), MMP-9 (polyclonal rabbit anti-human MMP-9, 1:100, NeoMarkers, Fremont, CA, USA, RB-9234-P0), and MMP-10 (polyclonal rabbit anti-human MMP-10, 1:100, NeoMarkers, RB-9235-P0) for one hour at room temperature. The stainings were performed with the ZytoChem-Plus AP Kit (Broad Spectrum, AP060, Zytomed, Berlin, Germany) and the Alkaline Phosphatase Substrate Kit I (Sk-5100, Vector, Peterborough, UK) was used as detection system. The slices were counterstained with Mayer’s Hematoxylin. Negative controls were made using the secondary antibody only, as well as an isotope control staining.

### 4.5. Statistics

Statistical analysis was performed using SPSS 20 (IBM, Armonk, NY, USA). Data are presented as boxplots with median and 25% and 75% percentiles. The Kruskal–Wallis Test was used to determine significant differences between the four groups (one intact tendon group and three groups at different post rupture time-points). The Mann–Whitney *U*-Test was used to evaluate differences between two groups. Bonferroni–Holm Correction was used to adjust the *p*-value for two sets of three group comparisons (intact versus three ruptured groups and ruptured groups with each other). Statistical significances are given as exact significances. Additionally, for the gene expression analysis, a Spearman’s Rho correlation (r_s_) analysis was performed.

## 5. Conclusions

This is the first study utilizing different time-points of surgical intervention after acute rupture to characterize the biological environment at the rupture site during tendon healing, regarding structural tendon changes and the expression of important matrix-degrading enzymes and their inhibitors. This study demonstrates a pivotal contribution of all investigated MMPs, as well as TIMP-1, but a minor role of TIMP-2, -3, and -4, during the early human tendon healing process.

## Figures and Tables

**Table 1:** Demographic parameters.

Group	Age Mean ± SD	Sex (Male/Female)	BMI Mean ± SD	Smoking (Yes/No)
Early	39.7 ± 10.3	14/1	25.6 ± 3.7	3/12
Middle	37.0 ± 9.0	12/2	24.9 ± 2.6	4/10
Late	34.6 ± 11.9	8/0	25.1 ± 2.1	2/6
Intact	38.7 ± 15.5	2/1	32.0 ± 3.3	1/2

BMI: body mass index.

**Table 2:** Classification of histological score.

Characteristic	Staining	Classification			
		0	1	2	3
Tendon architecture	MP	Parallel aligned, tightly packed collagen bundles	Bundles separated but aligned fibers still visible	Loss of bundles, fibers still parallel	Complete loss of tendon architecture
Aligned collagen	MP	76–100%	51–75%	26–50%	0–25%
GAG content	MP	No GAG content	Few small GAG areas	Large GAG areas	GAG-rich in large parts of the tendon
Fat content	MP	No fat	<5%	5–25%	>25%
Cellularity	H&E	No cell clusters	Sporadic small cell clusters primarily at boundaries	Several large cell clusters	High cellularity over the entire tendon tissue
Vascularity	αSMA	Blood vessels only between bundles	Sporadic blood vessels within the tissue	Several blood vessel clusters	Numerous blood vessels/clusters

**Table 3:** qRT-PCR Primer.

Gene	Accession No.	Primer Sequence	Size (bp)
18S RNA	NM_022551	Forward: 5′CGGAAAATAGCCTTTGCCATC3′ Reverse: 5′AGTTCTCCCGCCCTCTTGGT3′	107
MMP1	NM_002421.3	Forward: 5′CACGCCAGATTTGCCAAGAG3′ Reverse: 5′GTCCCGATGATCTCCCCTGA3′	148
MMP2	NM_004530	Forward: 5′TGGATGATGCCTTTGCTCGT3′ Reverse: 5′CCAGGAGTCCGTCCTTACCG3′	156
MMP3	NM_002422.3	Forward: 5′TGGGCCAGGGATTAATGGAG3′ Reverse: 5′GGCCAATTTCATGAGCAGCA3′	104
MMP9	NM_004994.2	Forward: 5′GGGACGCAGACATCGTCATC3′ Reverse: 5′GGGACCACAACTCGTCATCG3′	150
MMP10	NM_002425	Forward: 5′CCACCTGGACCTGGGCTTTA3′ Reverse: 5′GAACTGGGCGAGCTCTGTGA3′	192
MMP13	NM_002427.3	Forward: 5′CCTTCCCAGTGGTGGTGATG3′ Reverse: 5′CGGAGCCTCTCAGTCATGGA3′	144
TIMP1	NM_003254.2	Forward: 5′TTGGCTGTGAGGAATGCACA3′ Reverse: 5′AAGGTGACGGGACTGGAAGC3′	128
TIMP2	NM_003255.4	Forward: 5′CCTGAGCACCACCCAGAAGA3′ Reverse: 5′TCCATCCAGAGGCACTCGTC3′	123
TIMP3	NM_000362.4	Forward: 5′CCGAGGCTTCACCAAGATGC3′ Reverse: 5′GCCATCATAGACGCGACCTG3′	140
TIMP4	NM_003256.3	Forward: 5′GAAGCCAACAGCCAGAAGCA3′ Reverse: 5′TTCCCTCTGCACCAAGGACA3′	120
COL1A1	NM_000088.3	Forward: 5′TGACCTCAAGATGTGCCACT3′ Reverse: 5′ACCAGACATGCCTCTTGTCC3′	197
COL3A1	NM_000090.3	Forward: 5′GCTGGCATCAAAGGACATCG3′ Reverse: 5′TGTTACCTCGAGGCCCTGGT3′	199
